# Eco-hydrological modeling of soil wetting pattern dimensions under drip irrigation systems

**DOI:** 10.1016/j.heliyon.2023.e18078

**Published:** 2023-07-09

**Authors:** Dinesh Kumar Vishwakarma, Rohitashw Kumar, Arvind Singh Tomar, Alban Kuriqi

**Affiliations:** a Department of Irrigation and Drainage Engineering, Govind Ballabh Pant University of Agriculture & Technology, Pantnagar, Udham Singh Nagar 263145, India; b Department of Irrigation and Drainage Engineering, College of Agricultural Engineering and Technology, Sher-e-Kashmir University of Agricultural Sciences & Technology of Kashmir 190025 Jammu and Kashmir, India; c CERIS, Instituto Superior Técnico, University of Lisbon, 1649-004 Lisbon, Portugal; d Civil Engineering Department, University for Business and Technology, Pristina, Republic of Kosovo

**Keywords:** Drip irrigation, Empirical equations, Radial wetted radius, Vertical wetted depth, Wetting front coordinates, Wetting patterns

## Abstract

Reliable information on the horizontal and vertical dimensions of the wetted soil beneath a point source is critical for designing accurate, cost-effective, and efficient surface and subsurface drip irrigation systems. Several factors, including soil properties, initial soil conditions, dripper flow rate, number of drippers, spacing between drippers, irrigation management, plant root characteristics, and evapotranspiration, influence the dimensions and shape of wetting patterns. The objective of this study was to briefly review previous studies, collect the analytical, numerical, and empirical models developed, and evaluate the effectiveness of the most common empirical method for predicting the dimensions of soil wetted around drippers using measured data from field surveys. With this review study, we aim to promote a better understanding of soil water dynamics under point-source drip irrigation systems, help improve soil water dynamics under point-source drip irrigation systems, and identify issues that should be better addressed in future modeling efforts. A drip irrigation system was configured with three different emitters with different capacities (2, 4, and 8 l h-1) in the point source to determine the soil wetting front under the point source. The five most selected empirical equations (Al-Ogaidi, Malek and Peters, Amin and Ekhmaj, Li and Schwartzman and Zur) were statistically analyzed to test the efficiency in sandy loam soil. According to the results of the field investigation, statistical comparisons of the empirical models with the field investigation data were performed using the mean absolute error (MAE), root mean square error (RMSE), Nash–Sutcliffe model efficiency (CE), and coefficients of determination (R2). The advanced simulation of the wetting front was used based on the best accuracy of the selected empirical model. In general, the Li model (MAE, RMSE, EF, and R2 were 0.698 cm, 0.894 cm, 0.970 cm^2^ cm-2, and 0.970, respectively, for the wetted soil width and 1.800 cm, 1.974 cm, 0.927 cm^2^ cm-2, and 0.986, for the vertical advance) proved to be the best after statistical analysis with field data.

## 1 Introduction

In India, agriculture is the sector that consumes most of the water resources (almost 80%) for environmental reasons [1]. As the population density is increasing daily and water consumption is increasing, the irrigation system needs to be modified to produce more with less water and more food, while the agricultural productivity of soil and water resources is suffering. Microirrigation technology is widely used and applied worldwide [2]. In areas where water resources are becoming scarce and problematic, drip irrigation technology leads to “more yield per drop of water” for more sustainable agriculture [3]. This approach is one of the most effective methods to deliver water and nutrients to the root zone of plants [4,5]. The water savings recorded compared to other surface irrigation methods could be as high as 50% for a drip irrigation system [6].

### 1.1 Review of previous investigations

Because of these advantages, drip irrigation technology has promoted its worldwide adaptation [7]. The main reason for water savings is that drip irrigation allows the soil to be moistened by about 30% compared to other irrigation systems [8] by reducing the moistened zone instead of the total irrigated area, thus reducing deep percolation, evaporation, surface runoff, and transport losses due to infiltration and percolation from the upper soil surface [9]. The fresh, moist part of the soil during drip irrigation, including the root zone of plants, is aware of the soil wetting pattern. The information on soil wetting pattern/moisture distribution pattern in a drip irrigation system for specific soil types and specific flow rates of drippers can be helpful for (a) the efficient/optimal design, operation, and management of a drip irrigation system, (b) the selection of an appropriate number of drippers, (b) selecting an appropriate number of drippers, (c) selecting an appropriate size of dripper flow rate to deliver optimal water and nutrient needs directly to the root zone, (d) determining the desired irrigation schedule, (e) matching soil wetting dimensions to the root pattern/zone of the crop [10]. The wetted soil dimensions, wetted soil volume, is a partially saturated zone within an elongated ellipsoid shape [11]. The dimensions of the wetted soil, the volume of the wetted soil, and the spatial redistribution of the soil moisture content during spot drip irrigation (above or below the soil surface) depend on several factors, such as. The pollution, physical properties of the soil, compaction, hydraulic properties of the soil, irrigation system characteristics (flow rates of drippers, (flow rates of drippers, spacing between drippers, irrigation frequencies, and their positioning above or below the soil surface), irrigation amount and interval, plant age, plant species, their root zone, plant water uptake, root growth patterns, and climatic conditions such as temperature, wind speed, and relative humidity [[12], [13], [14]].

Knowledge of the patterns of moisture distribution in infiltration systems for different soil types and different runoff rates is necessary: (a) to ensure efficient system design and operation, (b) to ensure precise placement of water and fertilizer in the active root zone, (c) to design irrigation schedules, and (d) to synchronize wetting with plant rooting [9]. The lack of knowledge about the spatial and temporal variations of soil moisture has led to inefficient water use in trickle systems because the system cannot be effectively managed [15]. The redistribution of soil water between drip and lateral lines should be homogeneous in properly installed and constructed drip irrigation systems [16]. Understanding the dimensions of wetting patterns is essential for reliable drip irrigation system design and efficient management of natural resource water use [16,17].

### 1.2 Factors affecting soil moisture dispersal under the surface and subsurface trickle irrigation

#### 1.2.1 Effect of soil texture on soil moisture distribution

As part of their research, Thorburn et al. [18] evaluated the wetting dimension of moistened soil under surface and subsurface drip irrigation based on the hydraulic properties of 29 soils representing a comprehensive data set of textures and soil properties to assess the influence and effect of soil texture and/or type on soil wetting patterns. They found that the soils belonged to two distinct groups that differed in their hydraulic properties as a function of soil texture and/or wetting dimension (i.e., the wetted radius was increased relative to the wetting depth below the emitter) — increasing the clay concentration of the first group of soils resulted in a more spherical shape for the second group of soils, which had less expression of field structure, as is commonly believed. However, there was no relationship between wetting size and texture in the second group of soils, where the field structure was intact. The soil wetting patterns he studied under drip irrigation are shown in Table 1.

**Table 1:** Calculated wetted volume dimensions (Wetted depth and wetted radius, units are m) in the 18 soils from Group 2 after application of 1.65 and 6.6 L of water (at 1.65 l h^−1^) [19].

Statistical values	Surface				Subsurface					
	1.65 (t = 1 h)		6.6 l (t = 4 h)		1.65 (t = 1 h)			6.6 l (t = 4 h)		
	r	z_+_	r	z_+_	r	z_+_	z-	r	z_+_	z-
Mean	0.16	0.25	0.23	0.44	0.14	0.22	0.11	0.21	0.40	0.16
Maximum	0.33	0.35	52	0.58	0.27	0.29	0.26	0.43	0.56	0.40
Minimum	0.10	0.20	0.13	0.35	0.10	0.18	0.07	0.13	0.31	0.08
CV	36	16	41	17	0.29	0.17	0.41	0.34	19	48

Where t is time, z_+_ is vertical distance downward, z- is upward distance, and r is radial distance.

Cote et al. [20] found that the wetting pattern was elliptical. The wetted soil depth was more extensive than the wetted soil radius in subsurface drip irrigation. At a depth of 30 cm in sandy soil, 94% of the water was applied below the emitter. In silty soil, the wetting pattern was nearly spherical. The horizontal and vertical dimensions of the wetting front were measured at 28 and 20 cm, respectively, for sandy loam soil in sandy irrigated palms at irrigation rates of 1, 1.8, and 3.4 l h-1 [21].

The soil wetting patterns studied by Ainechee et al. [22] under irrigation sprinkling showed that the amount of water increases the wetted width and depth. The maximum wetted width and depth were found in sandy soils for all irrigation water volumes, followed by silty loam and clay soils. Siyal and Skaggs [23] found that soil texture has a greater influence on wetting geometry due to its relationship with soil hydraulic conductivity and water retention. Greater horizontal expansion in the wetted dimensions usually occurs in the fine-textured soils or the fine-textured layers of the layered soils. Nafchi et al. [24] studied the geometry and volume of the wetted soil during drip irrigation (Table 2). For soils with fine texture, the average diameter of the wetted soil volume was larger than for soils with coarse texture. The average diameter of wetted soil volume for fine textured soils was greater than for coarse-textured soils.

**Table 2:** Variation of wetted depth and width with soil texture for drip irrigation (Nafchi et al. [24]).

Soil texture	Applied emitter flow rate (l h^−1^)	Wetted soil diameter (cm)
Clay	2	49.12
	4	62.58
	8	66.50
	12	71.75
Loam	2	42.92
	4	49.08
	8	58.74
	12	68.07
Sandy Loam	2	22.29
	4	40.22
	8	48.11
	12	50.12

Yasin et al. [25] have shown that a slight increase in horizontal feed and an almost nonexistent increase in vertical feed enhance the changes in bulk density and soil depth while diagonally. They also showed that the increase in vertical advance is more significant than that in horizontal advance, that it can be continued diagonally by increasing the application rate, and that all horizontal advances, vertical advances, and diagonal advances of the wetting front increase when the same amount of water is applied with the decrease in application rate.

Fan et al. [26] investigated and showed that the geometry of the soil-wetting pattern exhibits slight but significant size changes. Moisture is generally “ellipsoidal” in its wetting pattern and shape and in the volumetric soil water content contour. Soil texture has a significant effect on the characteristics of the wetting patterns. The vertical and horizontal frontal wetting distance and soil moisture volume decrease as the clay concentration of the soil increases.

#### 1.2.2 Effect of the flow rate of dripper on soil moisture distribution

The radius of the wetted surface under spot drip irrigation on Malaysian sandy soil was studied by Ekhamaj et al. [27]. The study showed that soil moisture decreased with depth downward and horizontally from the point source of application because moisture was mainly maintained within 0.2 m of the point source. The wetted soil depth of all application rates was reported to be greater than the maximum wetted soil radius at the surface.

Thabet and Zayani [28] studied the wetting patterns in loamy, sandy soils under point source drip irrigation with two emitter flow rates, i.e., 1.5 and 4 l h-1. The study showed a higher vertical movement of soil moisture at a higher flow rate of 40 cm at 1.5 l h-1 after 6 h of water application. In contrast, it was 52.5 cm after 6 h of water application at 4 l h-1. However, the maximum wetting radius (30 cm) was recorded at the lower flow rate (1.5 l h-1) and the minimum (22 cm) at the higher delivery rate (3 h of application) (4 l h-1). Amer et al. [29] studied the geometric wetting of soil at different flow rates of 2, 4, 8, 16, and 24 l h-1 and different operating hours of irrigation from a point source of drip irrigation for cucurbits and grapes in an Egyptian district. The research study showed that the wetted soil depth almost doubled only after irrigation. However, the width of the wetted soil did not increase significantly. After water redistribution, the soil was almost twice as wide. Higher flow rates increased the width of soil wetting but decreased the wetted soil depth. Kandelous et al. [10] studied the distribution of soil water content between two SDI emitters. The study showed that the higher the flow rate of the emitters, the faster the horizontal wetting front progressed. However, the speed of the vertical wetting front is relatively unremarkable. Molavi et al. [30] recorded the coordinates of the soil-wetted bulb as the flow function of the emitters, time of water application, volumetric volume variation, and saturation of soil hydraulic conductivity. Saxena et al. [31] investigated that the maximum radius of wetted soil at and below the soil surface increased with the flow rate of the emitter. However, the wetted soil depth showed the same trend for Vertisols.

#### 1.2.3 Effect of the flow rate of dripper on soil moisture distribution

The wetted soil's width and depth depend on the irrigation duration [32], and both increase with time. In clayey mixed sand soils, the wetted soil depth was greater than the wetted width. Kyada and Munjarappa [33] reported that wetted width increased at the beginning of irrigation in clayey soils. However, the increase was relatively slow. The change in moisture distribution patterns for soil (wetted width and wetted soil depth) is summarized in Table 3 as a function of irrigation duration in different agroecological situations.

**Table 3:** Various wetted front advances with different irrigation duration for drip irrigation after Kyada and Munjarappa [33].

Irrigation duration (h)	Flow rate (l h^−1^)	Wetted width (cm)	Wetted depth (cm)	Location and soil type	Reference
3.2	4	16	17	Pakistan, loamy sand mixed	Phull and Babar [32]
4.2	4	17	19		
6	4	20	23		
1	2	12	36	India, clay loam soil	Kyada and Munjarappa [33]
2	2	23	41		
3	2	31	48		
4	2	36	52		
5	2	44	56		
1	4	26	44		
2	4	28	50		
3	4	38	52		
4	4	45	55		
5	4	55	60		
0.166	4	9.1	3.21	India, sandy loam soil	Reddy et al. [34]
1.23	4	21.67	17.56		
1.5	4	26.12	24.44		
2	4	26.12	24.44		
2.33	4	28.22	27.5		

### 1.3 The main research aims and constraints of this paper

The purpose of this study is to evaluate and summarize the various models that have been developed to determine the wetting process resulting from drip irrigation in the hope of achieving the specific objectives: (a) delineate between analytical, semi-analytical, numerical, and empirical models for determining the wetting pattern resulting from drip irrigation; (b) improve understanding of the soil moisture distribution pattern as a function of soil hydraulic properties, drip runoff rates, spacing, irrigation amounts and frequency, plant water uptake rates, and root distribution patterns, and provide planners with current information on the subject; (c) a brief compilation of existing models for estimating soil wetting geometry; (d) a compilation of different wetting fronts under different irrigation durations in drip irrigation; (e) an estimate of the wetting pattern in clay soils under a spot drip irrigation; and (f) an evaluation of the accuracy of five commonly used empirical models, namely Al-Ogaidi, Malek and Peters, Amin and Ekhmaj, Li and Schwartzman, and Zur with observed field studies.

## 2 Method and theoretical consideration

### 2.1 Description of various models

Researchers have developed several methods that define infiltration from a point/line source of drip irrigation and use them to predict wetting dimension patterns of the moistened soil (above or below the soil surface) for efficient, effective, economical, and accurate design, operation, and management of drip irrigation systems [35,36]. There are three main classes of models for evaluating soil wetting dimensions, namely (i) analytical, (ii) numerical, and (iii) empirical. Analytical models are developed in the mathematical models with a very closed form of a solution; that is, a mathematical, analytical function can explain how the equation of a system can change the solution. A computer model was created to simulate and replicate the mechanisms of a particular system. Empirical modeling is a collective term for practices that involve observations and experiments to create models. An empirical simulation is a form of simulation in which prototypes are developed using specific concepts. Analytical, empirical, and numerical models can help estimate soil moisture movement in the point source of drip irrigation for a given soil type, environmental setting, and various design parameters. Therefore, they can save time and financial capital compared to field tests. This study mainly aimed to collect the developed empirical analytical and numerical models and computer-coded programs to predict the coordinate of the wetting front. Several empirical equations were collected in the study.

### 2.2 Numerical models

Several models use the basic Richards equations as the governing equations of the water flow equation through porous media in the two-dimensional (2D) form to predict soil water pressure or moisture content distribution in unsaturated soil [37]. The hydraulic conductivity of soil under unsaturated movement conditions is nonlinear [38]. It depends on soil water pressure, converted to soil water content using water retention functions. Sometimes numerical flow models are considered inefficient and impractical for planning purposes. The following equation (1), an adapted form of Richard's equations, can be used to explain axisymmetric flow in a variably saturated, rigid, isotropic porous medium:

(1) $$\frac{\partial \theta }{\partial t}=\frac{\partial }{\partial s}\left(K\left(h\right)\frac{\partial h}{\partial s}\right)+\frac{K\left(h\right)}{s}\frac{\partial h}{\partial s}+\frac{\partial }{\partial z}\left(K\left(h\right)\left[\frac{\partial h}{\partial z}+1\right]\right)$$

where, θ = volumetric soil water content (L^3^/L^3^); h = soil pressure head (L); t = time (T); s = radial coordinate (L); z = vertical coordinate (L); K = unsaturated soil hydraulic conductivity (L/T).

Many researchers have studied and solved the above formula both numerically and analytically. In solving this equation, choosing the limits and initial conditions is crucial. Philip [39] and Gardner and Mayhugh [40] presented a new equation for Richard's equation, which they recommended use by solving the hydraulic conductivity (Eq. (2)).

(2) $$K\left(h\right)=K_{s}\;\exp\left(\alpha h\right)$$

where, K_s_ (L/T) = saturated soil hydraulic conductivity; α = reciprocal of macroscopic capillary length scale [41].

With the help of “WetUp,” a user-friendly computer program, the results presented by Philip [39] were used to compute the sizes of soil-wetting patterns [42].

### 2.3 Analytical models

These models are used to predict dimensions of soil wetting under the point source of drip irrigation, usually by solving the governing equation of the water flow equation under certain conditions. Cook et al. [42] developed the Microsoft Windows OS-based software program “WetUp” (v1.5) to perform the visualization of wetting patterns. It is user-friendly, environmentally friendly, and freely available to users. The coded program estimates the dimensions of soil wetting patterns in different soil textures with different physical and hydraulic soil properties for surface or subsurface (buried) point sources (emitters). The software includes a database of preconfigured soil types, dripper flow rates (from 0.503 to 2.7 l h-1), irrigation durations (from 1 to 24 h), initial soil moisture conditions (preset by 3, 6, and 10 m suction heights), and dripper location (above or below ground). Philip's [39] approach simulates runoff from an above- and below-ground point source in WetUp. The approach defines the water travel time based on a quasi-linear analysis of a constant three-dimensional unsaturated water flow. The distance between the buried source and the wetted perimeter at a given time can be calculated with equation (3) using Philip's equation (30) [39].

(3) $$T\left(R,\theta \right)=\frac{\exp\left(2R\;\sin^{2}\frac{1}{2}\emptyset \right)}{2\;\cos^{2}\frac{1}{2}\emptyset }\left\{R^{2}-R+\left[\ln\left(\cos\frac{1}{2}\emptyset \right)-R\;\sin^{2}\frac{1}{2}\emptyset +\frac{1}{2}\right].\ln\left(\frac{\exp\left[2R\;\sin^{2}\frac{1}{2}\emptyset \right]-\cos^{2}\frac{1}{2}\emptyset }{\sin^{2}\frac{1}{2}\emptyset }\right)-\frac{1}{2}\left[L\left(\sec^{2}\frac{1}{2}\emptyset \exp\left[2R\;\sin^{2}\frac{1}{2}\emptyset \right]\right)-L\left(\sec^{2}\frac{1}{2}\emptyset \right)\right]\right\}\;\left(0<\emptyset <\pi \right)$$

where r and ∅ denote the polar coordinates of a sphere (s = r sin∅, z = r cos∅), R = αr/2 in which α denotes the reciprocal of the macroscopic capillary length scale proposed by White and Sully [41], and L(x) denotes the dilogarithm defined by White and Sully [41] in Eq. (4):

(4) $$L\left(x\right)=-\int_{1}^{x}\frac{\ln\;x}{x-1}dx$$

where x is a dummy variable, and the dimensionless time T is given by Eq (5):

(5) $$T=\frac{\alpha^{3}\;qt}{16\;\pi \Delta \theta }$$

Where q is the emitter flow rate, t is irrigation time, Δθ is the average change in volumetric soil water content in the wetting zone of soil, and α is the reciprocal of the macroscopic capillary length scale. For a surface source, T is related to the wetted perimeter by equation (44) of Philip [39]:

(6) $$T\;\left(R,\emptyset \right)=\exp\left[R\left(1-\cos\emptyset \right)\right]\times \left\{R^{2}\left(1-\frac{1}{2}\cos\emptyset \right)-R+\frac{1}{\cos\emptyset }\left[R\left(1-\cos\emptyset \right)\ln\left(1-\cos\emptyset \right)+\ln\left(\frac{1-\cos\emptyset \exp\left[R\;\left(\cos\emptyset -1\right)\right]}{1-\cos\emptyset }\right)-L\;\left(1-\cos\emptyset \exp\left[R\;\left(\cos\emptyset -1\right)\right]+L\;(1-\cos\emptyset \right)\right]\right\}\left(0<\emptyset <\frac{1}{2}\pi \right)$$

In emerging the theory, we deliberate a source of strength q (L^3^ T^−1^) positioned at (s, z) = (0, 0). The radiated distance in the source plane (z = 0) and the maximum vertical distance (s = 0). Eqs. (3), (6) successfully solved to get estimates of R for a given set of T values using Maple 7 algorithms that were created in the MATLAB programming language [43]. A binary splitting process was used to reduce the relative difference [(LHS-RHS)/LHS] between the left and right halves of equations (3), (6) to less than 1 × 10^−6^, as described in the description of this method.

WetUp has been compared to other empirical and numerical solutions for approximating soil wetting dimensions [44]. Its predictions of the geometry of the soil-wetting pattern were less accurate than the empirical equation of Amin and Ekhmaj [45]. The results of the numerical model developed using Hydrus-2D by Simunek et al. [46]. Cook et al. [47] also reported that WetUp underestimated lateral soil wetting at large applied water volumes, especially for soils with coarse textures.

### 2.4 Empirical model

Several empirical models have been developed based on assumptions and drip conditions [48]. Interpretations from field observations have been combined with linear or nonlinear regression and dimensional analysis to create empirical models that can be tested for accuracy [49,50]. The rationale of the empirical models was expressed to estimate one of the parameters such as soil moisture content, the radius of wetted soil bulb, volume of wetted soil bulb, depth of soil wetting, boundaries and shape of wetted soil volume, which is a function of the total amount of water applied, irrigation measure, soil porosity, and soil physical and hydraulic properties (soil bulk density, percent sand, silt, and clay, average change in soil water content, initial soil moisture content, saturated soil hydraulic conductivity, or steady-state infiltration rate) using regression analysis (linear and nonlinear), dimensional analysis, and artificial neural networks (ANNs) from field investigations [2,51].

These methods are used when there is no knowledge of the hydraulic properties of the soil, and mathematical simplicity is essential. Some simple models have been developed for sizing soil-wetting patterns based on volume balance and flow geometry [52,53]. The empirical models developed in the past for surface and subsurface drip irrigation are listed in Table 4, Table 5 with their input parameters for estimating soil wetting.

**Table 4:** Different wetting pattern models for surface drip irrigation.

S. No.	Model(s)	Model equation	Parameters used
1.	Ben-Asher et al. [54]	R=(3qtΔθ)13,whereΔθ=θs2	Emitter flow rate, time duration of irrigation, and average change of soil moisture content.
2.	Schwartzman and Zur [52]	$W={1.28\;V}^{0.22}{\left(\frac{K_{s}}{q}\right)}^{-0.17}$ $Z={2.54\;V}^{0.63}{\left(\frac{K_{s}}{q}\right)}^{0.45}$	Emitter flow rate, the total volume of water applied, and Saturated hydraulic conductivity.
3.	Warrick [55]	$V_{s}=7.83\;V^{0.994}$ $W=2.9\;Z^{0.652}$	Total added water volume and wetted soil volume.
4.	Zur [53]	$V_{S}=\frac{\pi }{12}W^{2}\left[2Z+h-\frac{h^{3}}{{\left(Z-h\right)}^{2}}\right]$	Wetted soil width, depth, and depth between maximal lateral front advance and soil surface level.
5.	Li et al. [56]	$R=13.85\;{\left(q.t\right)}^{0.29}$ $Z=8.69\;{\left(q.t\right)}^{0.53}$	The total volume of applied water.
6.	Li et al. [57]	a. Sandy Soil $R=13.40\;{\left(q.t\right)}^{0.33}\text{,}$ $Z=12.10\;{\left(q.t\right)}^{0.46}$ b. Loamy Soil $R=13.60\;{\left(q.t\right)}^{0.29}\text{,}$ $Z=10.10\;{\left(q.t\right)}^{0.44}$	The total volume of applied water.
7.	Ekhmaj et al. [27]	a. For 3 l h^−1^, $R=0.89V^{0.29}$ b. For 5.616 l h^−1^, $R=0.91V^{0.28}$ c. For 6.984 l h^−1^, $R=0.88V^{0.29}$ d. For any rate (l h^−1^), $R=0.90V^{0.28}$	Flow rate and total volume of applied water.
8.	Amin and Ekhmaj [58]	$R={\Delta \theta }^{-0.5626}V^{0.2686}q^{-0.0028}{K_{s}}^{-0.0344}$ $Z={\Delta \theta }^{-0.383}V^{0.365}q^{-0.101}{K_{s}}^{0.195}$	The average change in volumetric water content within the wetted zone, the total volume of applied water, emitter flow rate, and saturated soil hydraulic conductivity.
9.	Acar et al. [59]	$\frac{x^{2}}{a^{2}}+\frac{{\left(z+h\right)}^{2}}{b^{2}}=1$ $x=\pm \frac{a}{b}\sqrt{b^{2}-{\left(z+h\right)}^{2}}$ $V_{oz}=\frac{a^{2}\pi }{{3b}^{2}}\left(b+h\right)\left({2b}^{2}-h^{2}+hb\right)$	Wetted soil volume, maximal lateral wetting front advance, depth between maximal lateral wetting front advance and vertical wetting front advance, and depth between maximal lateral front advance and soil surface level.
10.	Ainechee et al. [22]	a. Sandy soil $W=5.32V^{0.21}{\left(\frac{K_{s}}{q}\right)}^{0.185}$ $Z=8.04V^{0.59}{\left(\frac{K_{s}}{q}\right)}^{0.385}$ b. Loam soil $W=2.21\;V^{0.75}{\left(\frac{K_{s}}{q}\right)}^{0.625}$ $Z=2.23V^{0.52}{\left(\frac{K_{s}}{q}\right)}^{0.28}$ c. Silty clay loam soil $W=3.25V^{0.48}{\left(\frac{K_{s}}{q}\right)}^{0.22}$ $Z=4.06V^{0.74}{\left(\frac{K_{s}}{q}\right)}^{0.61}$	Emitter flow rate, the total volume of water applied, and Saturated hydraulic conductivity.
11.	Malek and Peters [60]	$R=q^{0.543}{K_{s}}^{0.772}t^{0.419}{\Delta \theta }^{-0.687}{\rho_{b}}^{0.305}$ $Z=q^{0.398}{K_{s}}^{0.208}t^{0.476}{\Delta \theta }^{-1.253}{\rho_{b}}^{0.445}$	Emitter flow rate, saturated hydraulic conductivity, time duration of irrigation, the average change in volumetric water content within the wetted zone, and bulk density of soil.
12.	Molavi et al. [30]	a. For 2 l h^−1^ $W=4.5327\;{K_{s}}^{0.03765}t^{0.5187}$ b. For 4 l h^−1^ $W=1.9038\;{K_{s}}^{–0.1282,}t^{0.58248}$	Saturated hydraulic conductivity and time duration of irrigation.
13.	Samadianfard et al. [61]	$R=20.6+\left[\left(29.4\;\theta_{r}\right)-\left(15.7\;\theta_{s}\right)-\left(15.7\;\theta_{i}\right)-\left(206\;\alpha \right)-\left(0.28\;n\right)+\left(0.00605\;K_{s}\right)+\left(5.4\;q\right)+\left(3.36\;t\right)\right]$ $Z=19.2+\left[\left(8.8\;\theta_{r}\right)-\left(61.3\;\theta_{s}\right)+\left(74.3\;\theta_{i}\right)-\left(105\;\alpha \right)-\left(6.15\;n\right)+\left(0.0744\;K_{s}\right)+\left(8.52\;q\right)+\left(5.96\;t\right)\right]$	Physical properties of soil, emitter flow rate, and duration of irrigation.
14.	Zhang et al. [62]	$W=14.69{\left(q.t\right)}^{0.35}$ $Z=7.9{\left(q.t\right)}^{0.56}$	Emitter flow rate and time duration of irrigation.
15.	Krada and Munjapara [33]	$W=28.9308\;q^{0.1283}t^{0.8137}$ $Z=18.3943\;q^{0.4996}t^{0.1954}$	Emitter flow rate and time duration of irrigation.
16.	Ismail et al. [63]	a. Continuous flow $W=0.769\;K_{s}^{0.1945}t^{0.463}q^{0.2685}$ $Z=0.887\;K_{s}^{0.6325}t^{0.755}q^{0.1225}$ b. Intermittent flow $W=1.202\;K_{s}^{-0.2}t^{0.2}q^{0.4}{CR}^{0.369}$ $Z=1.183\;K_{s}^{0.196}t^{0.465}q^{0.267}{CR}^{0.295}$	Saturated hydraulic conductivity, emitter flow rate, and time duration of irrigation.
17.	Naglič et al. [64]	$W={1.56\;V}^{0.29}{\left(\frac{K_{s}}{q}\right)}^{-0.057}$ $Z={1.71\;V}^{0.41}{\left(\frac{K_{s}}{q}\right)}^{0.11}$	Emitter flow rate, the total volume of water applied, and Saturated hydraulic conductivity.
18.	Al-Ogaidi et al. [65]	$R={7.0916\;q^{0.2562}t}^{0.2717}{K_{s}}^{2.0770}{\theta_{i}}^{0.1122}{\rho_{b}}^{-0.2435}S^{-0.1082}{S_{i}}^{0.0852}C^{-0.1540}$ $Z={0.4586\;q^{0.3902}t}^{0.3249}{K_{s}}^{6.1919}{\theta_{i}}^{0.0520}{\rho_{b}}^{0.0010}S^{-0.0928}{S_{i}}^{0.2574}C^{-0.2162}$	Emitter flow rate, duration of irrigation, soil bulk density, initial soil moisture content, saturated soil hydraulic conductivity, percentage of sand silt and clay.
19.	Al-Ogaidi et al. [66]	$R=0.0625\;t^{0.2562}q^{0.2716}\rho_{b}^{-0.0255}\theta_{i}^{0.1112}K_{s}^{0.335}S^{0.6303}{Si}^{0.1222}C^{0.6028}$ $Z=6.3555t^{0.3903}q^{0.324}\rho_{b}^{1.8315}\theta_{i}^{0.0198}K_{s}^{-0.084}S^{-0.1917}S_{i}^{0.1105}C^{-0.4265}$	Emitter flow rate, duration of irrigation, soil bulk density, initial soil moisture content, saturated soil hydraulic conductivity, percentage of sand silt and clay.
20.	Al-Ogaidi et al. [67]	a. Homogeneous profiles: $WP={a_{1}t}^{a1}q^{a2}{\rho_{b}}^{a3}{\theta_{i}}^{a4}{K_{s}}^{a5}S^{a6}{S_{i}}^{a7}C^{a8}$ b. Layered profiles $WP={b_{1}t}^{b1}q^{a2}{\left(\frac{\rho_{b1}}{\rho_{b}2}\right)}^{b3}{\left(\frac{\theta_{i1}}{\theta_{i}2}\right)}^{b4}{\left(\frac{K_{s1}}{K_{s2}}\right)}^{b5}{\left(\frac{S_{1}}{S_{2}}\right)}^{b6}{\left(\frac{S_{i1}}{S_{i2}}\right)}^{b7}{\left(\frac{C_{1}}{C_{2}}\right)}^{b8}$*Refer to the original paper for the empirical coefficient.*	Emitter flow rate, duration of irrigation, soil bulk density, initial soil moisture content, saturated soil hydraulic conductivity, percentage of sand silt and clay.
21.	Shan et al. [68]	$X_{if}=1.77t^{0.6358}\left(t_{1}\leq t\leq t_{2}\right)$ $X_{if}=1.06\;Z_{if}$	Intersection time of wetting front and final time of wetting front.
22.	Abu-Awwad et al. [69]	$WA=\frac{Q}{I}$	Emitter flow and infiltration rates at the time soil surface wetted area became almost constant.

**Table 5:** Different wetting pattern models for sub-surface drip irrigation.

S. No.	Model(s)	Model equation	Parameters used
1.	Philip [70]	$R_{s}=\left[\frac{2Q}{8\pi \Phi_{0}+\alpha Q}\right]$	Emitter flow rate, saturated hydraulic conductivity, and soil matric potential.
2.	Warrick [55])	$R_{0}=\frac{4}{\alpha }\exp\left[-\left(\frac{2\;\pi \;K_{s}}{\alpha .q}\right)-\gamma \right]$	Saturated bulb under steady-state conditions from a quasi-Emitter flow rate and saturated hydraulic conductivity.
3.	Shani and Or [71]	$R_{e}=\left[\frac{2Q\alpha }{8\pi K_{s}\left(\alpha P+1\right)+\alpha^{2}Q}\right]$	Saturated bulb under steady-state conditions from a quasi-Emitter flow rate, saturated hydraulic conductivity, and Soil water pressure head at the source.
4.	Singh et al. [36]	$W={3.27\;V}^{0.44}{\left(\frac{K}{q.z}\right)}^{-0.06}$ $Z={3.86\;V}^{0.31}{\left(\frac{K}{q.z}\right)}^{-0.19}$	Emitter flow rate, the total volume of water applied, and hydraulic conductivity of soil and emitter installation depth.
5.	Aldhfees et al. [72]	$Z={11.7\;V}^{0.63}{\left(\frac{K}{q}\right)}^{0.45}$	Emitter flow rate, the total volume of water applied, and Saturated hydraulic conductivity
6.	Kandelous et al. [73]	$W={4.244\;V}^{0.526}{\left(\frac{K_{s}}{q.z}\right)}^{-0.026}$ $Z_{+}={0.72\;V}^{0.344}{\left(\frac{K_{s}}{q.z}\right)}^{-0.156}$ $Z_{-}={0.66\;V}^{0.333}{\left(\frac{K_{s}}{q.z}\right)}^{-0.167}$	Emitter flow rate, the total volume of water applied, Saturated hydraulic conductivity, and emitter installation depth.
7.	Phull and Babar [32]	$W=3.245\;\left(\frac{q^{0.5}z^{0.065}t^{0.435}}{K_{s}^{0.065}}\right)$ $Z=3.572\;\left(\frac{q^{0.5}z^{0.177}t^{0.323}}{K_{s}^{0.177}}\right)$	Emitter flow rate, time duration of irrigation, Saturated hydraulic conductivity, and emitter installation depth.
8.	Liu et al. [74]	a. For the bulk density of 0.15 $W={0.379\;V}^{0.616}{\left(\frac{K_{s}\theta_{i}}{q.z}\right)}^{0.283}$ $Z={2.726\;V}^{0.216}{\left(\frac{K_{s}\theta_{i}}{q.z}\right)}^{-0.117}$ b. For the bulk density of 0.184 $W={0.151\;V}^{1.012}{\left(\frac{K_{s}\theta_{i}}{q.z}\right)}^{0679}$ $Z={2.957\;V}^{0.277}{\left(\frac{K_{s}\theta_{i}}{q.z}\right)}^{-0.056}$	Emitter flow rate, Total volume of applied water, initial soil moisture content, Saturated hydraulic conductivity, and emitter installation depth.
9.	Rasheed and Abid [75]	a. For sandy soil $W=7.599\;t^{0.471}{K_{se}}^{0.433}L^{0.354}h^{0.335}\theta^{0.099}$ $Z=16.891\;t^{0.396}{K_{se}}^{0.275}L^{0.521}h^{0.208}\theta^{0.108}$ b. For sandy Loam $W=6.301\;t^{0.481}{K_{se}}^{0.290}L^{0.314}h^{0.341}\theta^{0.137}$ $Z=13.493\;t^{0.229}{K_{se}}^{0.126}L^{0.470}h^{0.123}\theta^{0.086}$	Pressure head, saturated hydraulic conductivity, time duration of irrigation, soil moisture content, and length of ceramic pipe.
10.	Abid and Abid [76]	a. For 14.59 lph $W=22.4256\;t^{0.4551}q^{0.3318}{\theta_{i}}^{0.1284}z^{0.02}$ $Z=22.1315\;t^{0.4635}q^{0.3628}{\theta_{i}}^{0.1323}z^{0.0666}$ b. For 4.42 lph $W=22.2614\;t^{0.4722}q^{0.3515}{\theta_{i}}^{0.1644}z^{-0.0023}$ $Z=22.1775\;t^{0.4377}q^{0.3443}{\theta_{i}}^{0.1445}z^{0.0078}$ c. For 4.42 lph $W=10.8094\;t^{0.4552}q^{0.1404}{\theta_{i}}^{0.3479}z^{-0.2377}$ $Z=10.1302\;t^{0.4143}q^{0.1359}{\theta_{i}}^{0.2819}z^{0.2309}$	Emitter flow rate, time duration of irrigation, initial soil moisture content, and emitter installation depth.
11.	Zandi et al. [77]	a. Silty clay loam $w=2.9158v^{0.4366}z^{0.4366}{\left(\frac{k}{q}\right)}^{0.3732}$ $Z_{+}=1.358V^{0.5033}z^{0.5033}{\left(\frac{k}{q}\right)}^{0.5066}$ $Z_{-}=3.358V^{0.7063}z^{0.7063}{\left(\frac{k}{q}\right)}^{0.9126}$ b. Sand $w=1.596v^{0.3006}z^{0.3006}{\left(\frac{k}{q}\right)}^{0.1012}$ $Z_{+}=1.358V^{0.2179}z^{0.2179}{\left(\frac{k}{q}\right)}^{-0.0642}$ $Z_{-}=2.1596V^{0.47923}z^{0.47923}{\left(\frac{k}{q}\right)}^{0.4584}$ c. Loam $w=1.751v^{0.322}z^{0.322}{\left(\frac{k}{q}\right)}^{0.164}$ $Z_{+}=0.8879V^{0.3083}z^{0.3083}{\left(\frac{k}{q}\right)}^{0.1166}$ $Z_{-}=1.482V^{0.4247}z^{0.4247}{\left(\frac{k}{q}\right)}^{0.3546}$	Emitter flow rate, the total volume of water applied, and hydraulic conductivity of soil and emitter installation depth.

## 3 Study site

**Fig. 1:**
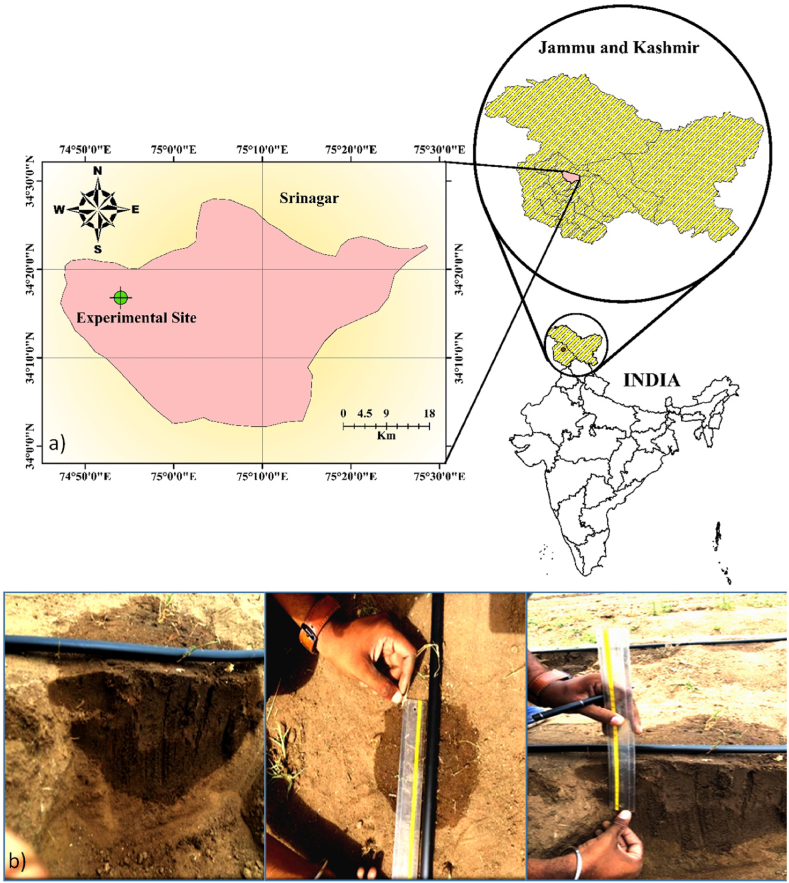
a) Study area of the experimental site and b) measurement of wetted soil front.

The experiment was conducted on an experimental farm of a high-density apple orchard of Sher-e-Kashmir University of Agricultural Sciences & Technology of Kashmir, Shalimar Campus (74.87o E longitude, 34.14o N latitude and 1606 m above mean sea level) for observation and measurement of wetting front (Fig. 1a).

The farm was laid out with a gravity drip irrigation system with a 2, 4 and 8 l h-1 flow rate. The most important and durable soil property is the texture of the wetting front. In order to compare the results of the empirical models for the simulated dimensions of the wetting patterns with the results of other empirical models, an observation data set under drip irrigation is required. Soil samples were collected from the experimental field at 0–15, 15–30, 30–45, and 45–60 cm soil depths. The particle size distribution was analyzed using a sieve set and a calibrated Bouyoucos hydrometer according to the methodology proposed by Bouyoucos [78] and sieve analysis (Gee and Or, [79]) and is shown in Table 6.

**Table 6:** Soil characteristics in terms of physical, hydraulic, and chemical characteristics.

S. No.	Particulars	Soil depth (cm)			
		0–15	15–30	30–45	45–60
1.	Coarse sand (%)	2.28	2.18	1.29	1.95
2.	Fine sand (%)	47.72	43.82	46.71	52.05
3.	Silt (%)	40	46	46	38
4.	Clay (%)	10	08	06	08
5.	Bulk density (g/cm^3^)	1.33	1.52	1.61	1.62
6.	Particle density (g/cm^3^)	2.8	2.8	2.8	2.8
7.	Field capacity (%)	36	36	21	21
8.	Porosity (%)	44	44	37.5	37.5
9.	Saturated hydraulic conductivity (cm/hr)	1.85	1.85	1.80	1.80
10.	Infiltration rate (mm/hr)	12	12	14	14
Textural Classes		Loam	Loam	Sandy Loam	Sandy Loam
11.	Soil pH	5.36	6.05	6.54	6.45
12.	EC (dSm^−1^)	0.60	0.06	0.09	0.08

The system has three side channels, one for 2 l h-1, one for 4 l h-1, and one for 8 l h-1 emitter discharge rates; for each side channel, three emitters are used for three replicates, and the average value is taken for each. In addition, each emitter and the side ducts were placed far away from the other emitters to eliminate the possibility of other emitters interfering with the wetting front of each emitter. A cross-section of soil approximately 0.50 m wide and 0.50 m deep was sharply delineated and filed after 24 h of each irrigation and t. The width and depth of the wetted soil front in each section were measured (Fig. 1b).

## 4 New observations and models accuracy test

Knowledge of crop water requirements, wetting pattern, wetted bale size, and multiple spray devices is necessary for optimal irrigation practices. The performance of the five most popular empirical models (Al-Ogaidi [66], Malek and Peters [60], Amin and Ekhmaj [58], Li [57], and the model of Schwartzman and Zur [52]) is evaluated using the measured wetting front during field investigation in sandy loam soils. The study's results will be useful in drip irrigation design, especially in selecting lateral and manifold spacing, runoff rate, and application time.

An experimental test was conducted to measure the soil wetting front. Based on the data collected during the field investigation, the models based on the calculation of the dimensions of the wetting pattern using selected empirical models were tested. Some statistical criteria were used to evaluate and illustrate the performance of the existing empirical models. Some statistical criteria were used to evaluate and illustrate the performance of the existing empirical models. The performance indicators used were: the mean absolute error (MAE), the root mean square error (RMSE), the Nash- Sutcliffe model efficiency (EF), and R2. The following relationships were used to calculate the various statistical indicators as listed below [80,81]:

**Table:** 

Performance indicator	Range	Optimal Value
$MAE=\frac{1}{N}\left|\sum_{i=1}^{N}\left({Wetting\;Front}_{Foreasted,i}-{Wetting\;Front}_{Measured,i}\right)\right|$	0 to ∞	0
RMSE=(1N∑i=1N(WettingFrontForeasted,i−WettingFrontMeasured,i)2)12	0 to ∞	0
$CE=1-\frac{\sum_{i=1}^{N}{\left({Wetting\;Front}_{Measured,i}-{Wetting\;Front}_{Foreasted,i}\right)}^{2}}{\sum_{i=1}^{N}{\left({Wetting\;Front}_{Measured,i}-\bar{{Wetting\;Front}_{Measured,i}}\right)}^{2}}$	−∞ to 1	1
$R^{2}=\frac{{\left[\sum_{i=1}^{n}\left({Wetting\;Front}_{Measured,i}-\bar{{Wetting\;Front}_{Measured,i}}\right)\sum_{i=1}^{n}\left({Wetting\;Front}_{Foreasted,i}-\bar{{Wetting\;Front}_{Foreasted,i}}\right)\right]}^{2}}{\sqrt{\frac{\sum_{i=1}^{n}{\left({Wetting\;Front}_{Measured,i}-\bar{{Wetting\;Front}_{Measured,i}}\right)}^{2}\sum_{i=1}^{n}{\left({Wetting\;Front}_{Foreasted,i}-\bar{{Wetting\;Front}_{Foreasted,i}}\right)}^{2}}{{Wetting\;Front}_{Measured,i}}}}$	0 to1	1

Where N denotes the total number of observations; ${Wetting\;Front}_{Foreasted,i}$ denotes ith simulated/forecast wetting front values; $\bar{{Wetting\;Front}_{Foreasted,i}}$ denotes the mean value of simulated/forecast wetting front data; ${Wetting\;Front}_{Measured,i}$ = ith measured wetting front values and $\bar{{Wetting\;Front}_{Measured,i}}=$ denotes the mean value of measured wetting front data.

## 5 Results and discussion

### 5.1 Model accuracy

Comparing all empirical models, the five most popular empirical models, namely Al-Ogaidi, Malek and Peters, Amin and Ekhmaj, Li and Schwartzman, and Zur were selected and compared with the observed field investigation (model equations in Table 1, S. No. 2, 6, 8,11, and 18). This model was found to be the best in this investigation. The model was considered an ideal estimate if it had a lower RMSE, MAE, and CE value and a higher R2 value [48].

A summary of model performance, including statistical parameters such as MAE, RMSE, CE, and R2, is provided in Table 7, Table 8. Table 7, Table 8 lists the goodness-of-fit test indices for all treatments compared in the study for each of the selected empirical models. In contrast to the horizontal wetting front, good agreement was found between the simulated and measured horizontal wetting fronts. The Al-Ogaidi model showed MAE = 3.952, RMSE = 4.050, CE = 0.385, and R2 = 0.997. In contrast, the models of Malek and Peters, Amin and Ekhmaj, Li, Schwartzman, and Zur showed MAE = 5.782, 1.332, 0.698, and 6.193, respectively; RMSE = 6.118, 1.453, 0.894, and 6.240; CE = −0.403, 0.921, 0.970, and −0.459, respectively; and R2 = 0.965, 0.997, 0.997, and 0.997, respectively. In contrast to the vertical wetting front, Al-Ogaidi's model showed MAE = 9.796, RMSE = 10.388, CE = −1.035, and R2 = 0.995. In contrast, the models of Malek and Peters, Amin and Ekhmaj, Li, and Schwartzman, and Zur showed MAE = 5.296, 6.979, 1.800, and 3.892, respectively; RMSE = 6.435, 7.443, 1.974, and 4.529, respectively; CE = 0.219, −0.045, 0.927, and 0.613, respectively; and R2 = 0.995, 0.989, 0.986, and 0.872, respectively.

**Table 7:** Comparison of various empirical models with observed data sets for predicting wetting front horizontal advance (wetted width).

S. No.	Model(s)	Statistical criteria			
		MAE (cm)	RMSE (cm)	EF (cm^2^/cm^2^)	R^2^
1	Al-Ogaidi	3.952	4.050	0.385	0.997
2	Malek and Peters	5.782	6.118	−0.403	0.965
3	Amin and Ekhmaj	1.332	1.453	0.921	0.997
4	Li model	0.698	0.894	0.970	0.997
5	Schwartzman and Zur	6.193	6.240	−0.459	0.997

**Table 8:** Comparison of various empirical models with observed data sets for the wetting front vertical advance (wetted depth) prediction.

S. No.	Model(s)	Statistical criteria			
		MAE (cm)	RMSE (cm)	EF (cm^2^/cm^2^)	R^2^
1	Al-Ogaidi	9.796	10.388	−1.035	0.995
2	Malek and Peters	5.296	6.435	0.219	0.995
3	Amin and Ekhmaj	6.979	7.443	−0.045	0.989
4	Li model	1.800	1.974	0.927	0.986
5	Schwartzman and Zur	3.892	4.529	0.613	0.872

As a result of the statistical evaluation criteria, the Li model outperformed the selected empirical models with the lowest MAE, RMSE, and the highest NSE and R2 among the selected empirical models. The horizontal wetting front model, followed by the models of Amin and Ekhmaj, Al-Ogaidi, Malek and Peters, and Schwartzman and Zur, performed the best (Table 7). The Li model performed better than the other empirical models among the selected models in terms of its statistical criteria, i.e., lowest MAE and RMSE, and has the highest CE and R2 among the selected models, followed by the model of Schwartzman and Zur, the model of Malek and Peters, the model of Amin and Ekhmaj, and the model of Al-Ogaidi for the vertical wetting front (Table 8).

The efficiency of Li's empirical model was very high compared with all other empirical models (Table 7, Table 8). The empirical model was developed specifically for the clay soil. It contributes to a more accurate prediction of the wetted soil dimensions because the model considers the conditions in the clay soil and adjusts the relationship accordingly.

**Fig. 2:**
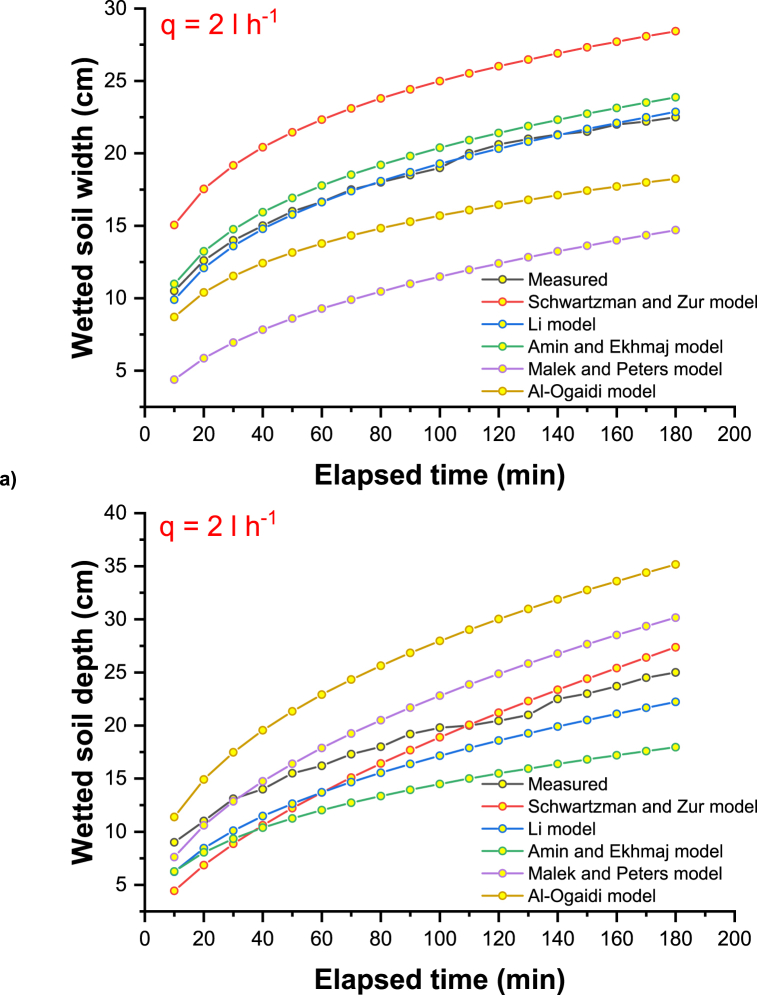

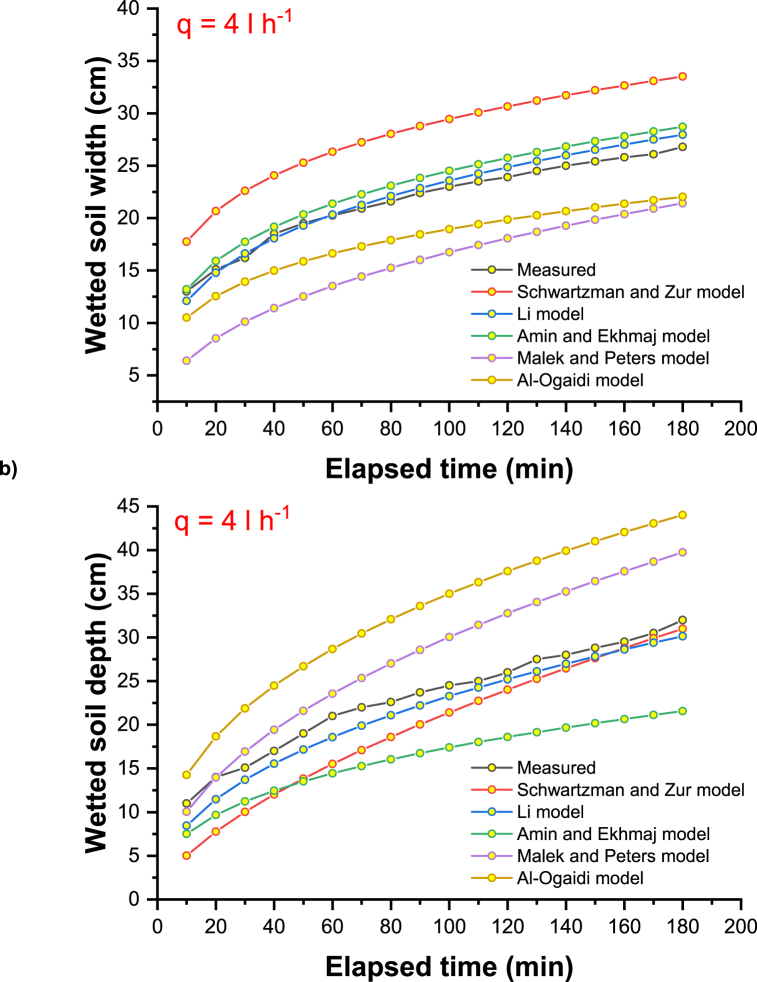

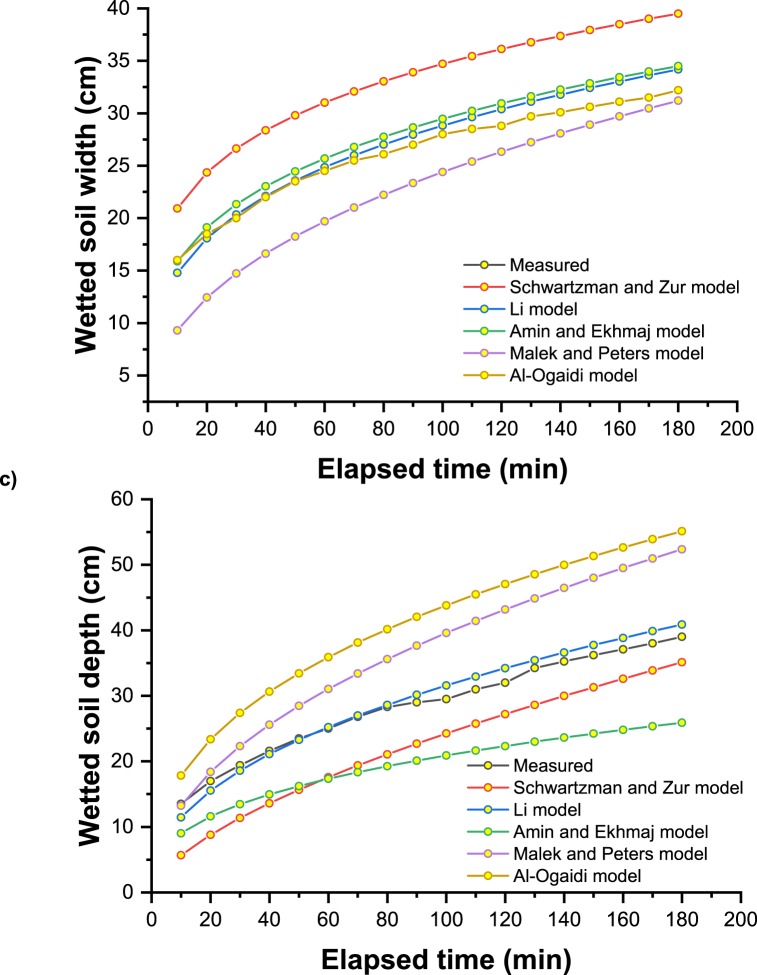
Measured and simulated wetting front advance under emitter discharge rate of a) 2 l h^−1^, b) 4 l h^−1,^ and c) 8 l h^−1^.

The main explanation for these results could be the consideration of the physical and hydraulic properties of the soil, such as the bulk and particle density, the percentage of clay, silt, and sand, the saturated hydraulic conductivity, and other optimistic field values such as the initial moisture content of the soil. Fig. 2a–c shows the measured and simulated propagation of the wetting front (horizontal and vertical) under the different discharge rates of the emitter (2, 4, and 8 l h-1). In Fig. 2, the difference between the value predicted by the model and the observed value using the model of Schwartzman and Zur is slightly larger for the initial time and slightly smaller for the wetted soil width. Compared to other empirical models, Schwartzman and Zur predicted the overestimation to a greater extent than other empirical models. For clayey soils, an inverse trend was observed for the depth of vertically wetted soil, and this trend is very similar to the all-emitter discharge rates with the measured vertical advance of a wetting front.

The radius and depth of soil surface wetting are slightly underestimated by the Li model, as shown in Fig. 2. Only minor discrepancies were found between the simulated and observed values, except for the model of Schwartzman and Zur, which underestimates the radius of surface wetting [60]. Considering the averaged distribution of plant roots, a designer could use these empirical relationships to find an appropriate flow rate and spacing of emitters. Then, the horizontal extent of the soil-wetting front could be easily determined.

### 5.2 Relationship between wetted soil width and depth

**Fig. 3:**
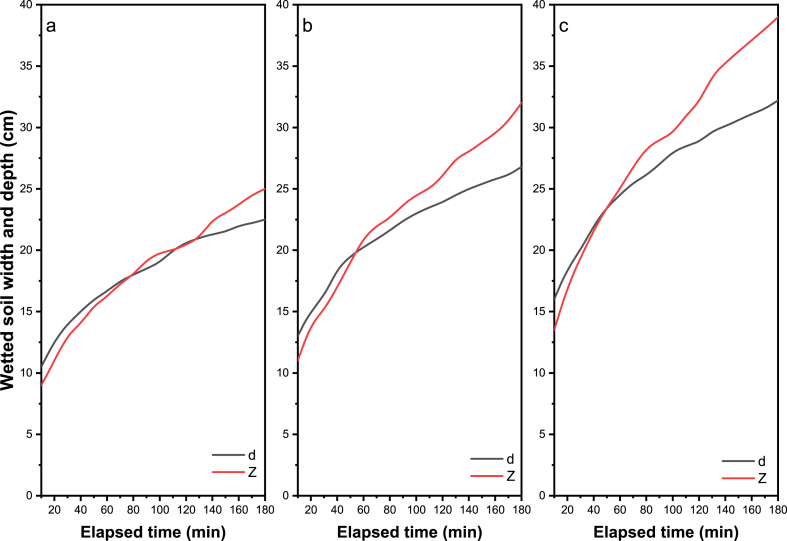
Measured wetting front dimensions against the elapsed times for all the experiments (a) 2 l h^−1^, (b) 4 l h^−1^, and (c) 8 l h^−1^. Note: d = wetted width and Z = wetted depth.

The width and depth of the wetted zones of the soil play an important role in the design of drip irrigation systems. Thus, for different irrigation durations, the wetted soil's width and the wetted soil's depth were determined for drippers with a capacity of 2, 4, and 8 l h-1. The following wetting curves are obtained from the field tests and the results obtained (Fig. 3a–c). The effect of different duration of irrigation and different flow rates of the emitters on the wetted dimensions using different flow rates of the emitters (i.e., 2, 4, and 8 l h-1) are shown in Fig. 3. The radius and depth of the wetted zone increased in all cases when the flow rate of the emitter increased in all cases.

Table 9, Table 10 summarize the effect of the different types of emitters on the measured wetted soil width or depth. As shown in Table 9, the width of the wetted soil was measured and was 14.65, 16.65, and 20.62 cm at an emitter discharge rate of 2 l h-1; rate: 16.20, 20.25, 23.90 cm at an emitter application rate of 4 l h-1; and 20.00, 24.50, 28.81 cm at an emitter application rate of 8 l h-1 with 30-, 60-, and 120-min application times. Similarly, in Table 10, the wetted soil depth was measured and was 13.10, 16.20, and 20.44 cm at an application rate of 2 l h-1; 15.10, 21.50, and 26.00 cm at an application rate of 4 l h-1; and 19.40, 25.00, and 31.00 cm at an application rate of 8 l h-1 with 30-, 60-, and 120-min application times, respectively.

**Table 9:** Average wetted soil width (cm) as influenced by different emitter flow rates and irrigation time from the point source of drip irrigation.

S. No.	Emitter flow rate (lph)	Wetted soil width (cm) from the point source of drip irrigation		
		Irrigation duration		
		30 Minutes	1 Hour	2 Hours
1.	2	14.65	16.65	20.62
2.	4	16.20	20.25	23.90
3.	8	20.00	24.50	28.81

**Table 10:** Average wetted soil depth (cm) is influenced by different emitter flow rates and irrigation time from the point source of drip irrigation.

S. No.	Emitter flow rate (lph)	Wetted soil depth (cm) from the point source of drip irrigation		
		Irrigation duration		
		30 Minutes	1 Hour	2 Hours
1.	2	13.10	16.20	20.44
2.	4	15.10	21.50	26.00
3.	8	19.40	25.00	31.00

**Fig. 4:**
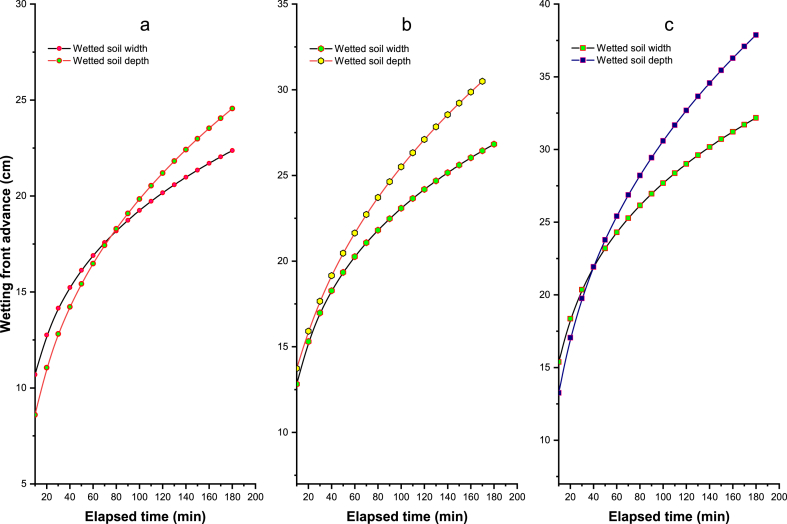
Simulated wetted width (d) and depth (Z) as a function of time for sandy loam soil at different: q = a) 2; b) 4 and c) 8 l h^−1^.

The width and depth of the wetted soil were predicted using Li models for different irrigation durations (2 l h-1, 4 l h-1, and 8 l h-1) (see Fig. 4). The similar increase in the radius of the wetted soil was initially rapid and then slowed over time with the measured value, as shown in Fig. 4. A similar trend was also observed for 4 l h-1 and 8 l h-1 emitter flow rates.

The predicted moist soil width 10 min after the start of irrigation was found to be 10.41 cm. The highest value after 3 h was 21.82 cm for 2 l h-1 emitter (Fig. 4a). Similarly, the wetted soil depth 10 min after the start of irrigation period was 7.95 cm, the maximum after 3 h, i.e. 24.56 cm, as shown in Fig. 4b.

In contrast, Fig. 4b shows that at a flow rate of 4 l h-1 of the emitter, the width of the wetted soil was 12.56 cm 10 min after the start of irrigation, and 3 h later, the maximum width of the wetted soil was 26.34 cm. Similarly, the depth of the wetted soil was 9.95 cm and 30.75 cm, respectively. From Fig. 4c, it can be seen that at a flow rate of 8 l h-1, after 10 min of irrigation, the width of the wetted soil was found to be 15.16 cm, and 3 h later, the maximum width of the wetted soil was 30.86 cm. Similarly, the depth of the wetted soil 10 min after irrigation was 12.46 cm, and maximum 36.76 cm after 3 h.

The higher the water applied, the more vertical wetting front was found. Similar results were found by Mostaghimi et al. [82]. When the irrigation duration was increased from 1 h to 5 h, and the flow rate was increased from 0.6 l h-1 to 3.0 l h-1, more vertical wetting of the soil was found than horizontal wetting front [83]. However, there was little progress in the vertical front when the irrigation duration and sprinkler flow rate increased. Therefore, irrigation duration needs to be improved to prevent water loss due to deep percolation, mainly with a long time scale for the higher flow rate and, for drip irrigation, mainly with a short time scale for the slightly lower flow rate. The relationship between the wetted soil front and the elapsed time at different flow rates of the emitters is shown in Fig. 4a-c.

**Fig. 5:**
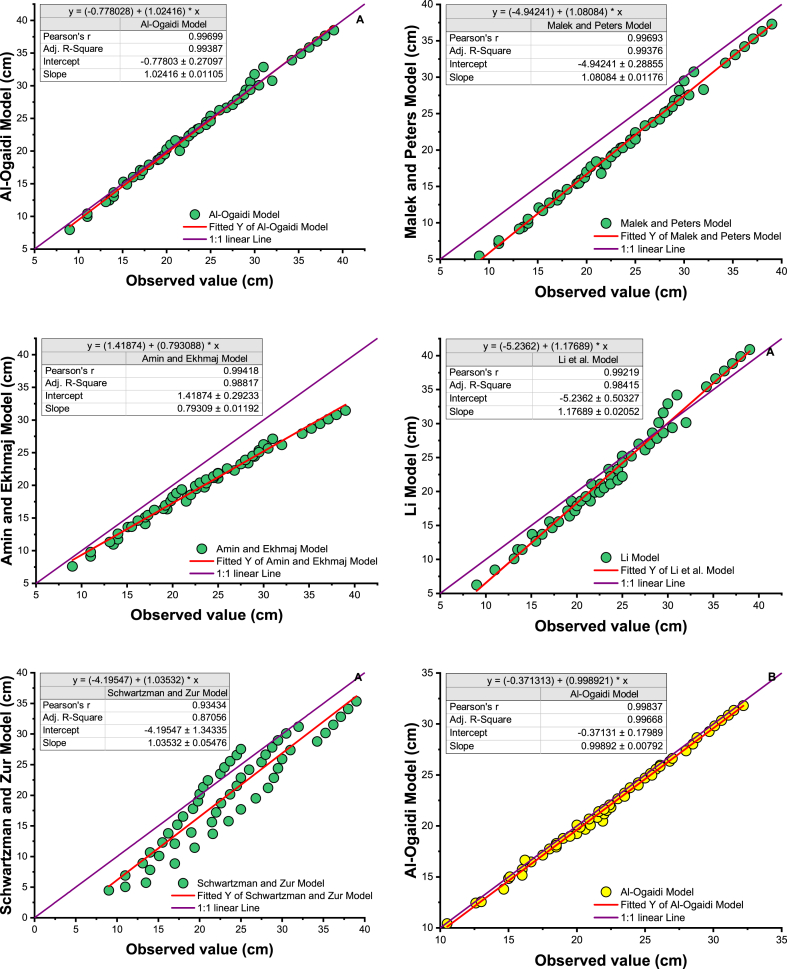

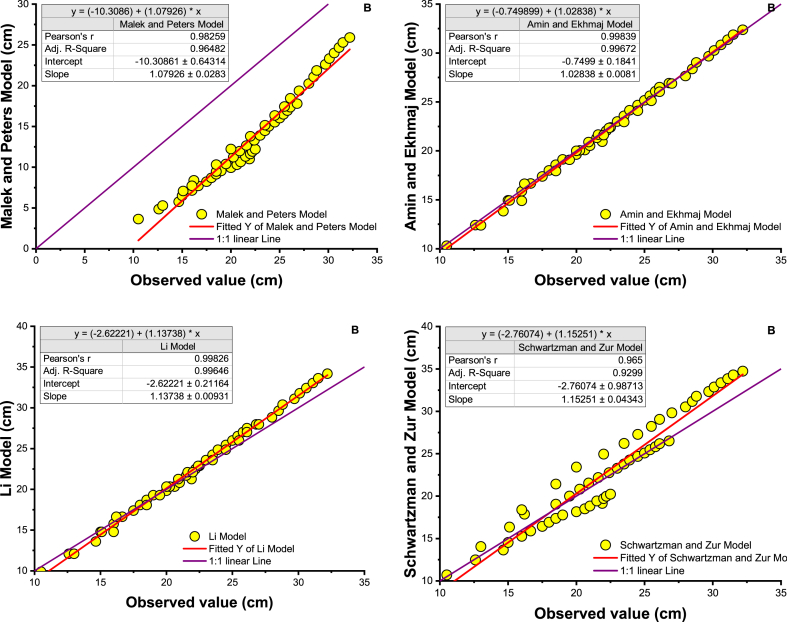
Scatter plot of measured vs. estimated wetted soil dimensions from the selected proposed model with line 1:1. A. wetted depth, B. wetted width.

In addition, the result of the models considered is illustrated by plotting the observed versus predicted wet dimensions of the soil for each model with a 1:1 axis (Fig. 5a). To illustrate the model results, a linear line was inserted in Fig. 5a, showing that the performance of the Li model, followed by the Al-Ogaidi, Malek, and Peters models, is reasonable because all points have a uniform distribution on a best-fit line (1:1) of wetted soil width. To illustrate the results of the model, a linear fit line was included in Fig. 5a, showing that the performance of the Li model, followed by the Amin and Ekhmaj and Al-Ogaidi models, is adequate since all points have a uniform distribution on a best-fit line (1:1) in the wetted soil depth. Malek and Peter's model was very influential in determining the advance of the wet front, especially at the wet depth, as shown in Fig. 5b. An empirical model by Schwartzman and Zur showed lower performance in determining wetted width and depth.

## 6 Conclusion

The distribution of soil water beneath a point source of drip is a three-dimensional problem. Accurate estimation of the wetted dimensions of the soil under drip irrigation can help designers determine the most efficient flow rates and spacing of emitters. The geometry of wetted soil dimensions depends primarily on the physical properties of the soil, drip irrigation parameters such as spacing of drip emitters, flow rate, number of drip emitters per plant or tree, amount of water applied, irrigation frequency, and plant evapotranspiration, and other factors. All of these factors must be considered when designing drip irrigation systems. Several analytical, numerical, and empirical models have been developed to predict the dimensions of wetted soil under a spot surface or subsurface irrigation system. These models are based on experimental results for different soil types and irrigation flow rates. They have yet to be calibrated and should be validated on other site-specific conditions. Wetting front patterns are generated using either an analytical or a numerical model. However, this method takes more time and is tedious and challenging.

Using the above different models, predicting the geometry of the wetted soil dimensions under certain conditions is beneficial when designing the drip irrigation system. The author selected five empirical models based on the most popular ones: i.e., Al-Ogaidi, Malek and Peters, Amin and Ekhmaj, Li & Schwartzman, and Zur models. It is very environmentally friendly and simple to determine the moisture geometry of the soil under spot drip irrigation.

Although no studies have yet been conducted under semiarid conditions to directly quantify the effects of these irrigation systems on tree water status, tree orchard water use, and fruit yield and quality over many years, future research can be conducted on these aspects. In addition to this study, further improvements can be made through practical integration with field work and additional modeling and irrigation scenario analysis to expand this work further. For better prediction accuracy, a wide range of emitter discharge rates should be analyzed better to predict emitter spacing under different irrigation system operating conditions. It is recommended that field trials be conducted to investigate if and how nitrogen and potassium move with the waterfront in the soil based on the timing of application and successive applications.

## Author contribution statement

Dinesh Kumar Vishwakarma and Rohitashw Kumar: Conceived and designed the experiments; Performed the experiments; Analyzed and interpreted the data; Wrote the paper. </p>

Arvind Singh Tomar, and Alban Kuriqi: Analyzed and interpreted the data; Contributed reagents, materials, analysis tools, or data; Wrote the paper. </p>

## Data availability statement

Data will be made available on request.

## Additional information

No additional information is available for this paper.

## Funding statement

The financial support was received through ICAR- AICRP on Plasticulture Engineering and Technology-sponsored projects during the experiment.

## Declaration of competing interest

The authors declare that they have no known competing financial interests or personal relationships that could have appeared to influence the work reported in this paper.

## Notations

WA: Soil surface wetted area (L^2^)
Q: Measured emitter flow rate due to pressure build-up in the soil surrounding emitters (L^3^T^−1^)
I: Infiltration rate at the time soil surface wetted area became constant (LT^−1^)
V_oz_: Wetted soil volume (L^3^)
a: ½ of the maximal lateral wetting front advance (L)
b: Depth between maximal lateral wetting front advance and vertical wetting front advance (L)
h: Depth between maximal lateral front advance and soil surface level (L)
W: Wetted soil width (L)
R: Wetted soil radius (L)
Z: Wetted soil depth (L)
K_s_: Saturated hydraulic conductivity (L^1^T^−1^)
V: Total volume of applied water (L^3^)
q: Emitter flow rate (L^3^T^−1^)
a_1_,..a^8^: Empirical coefficients (dimensionless)
WP: Any wetted parameter (L)
b_1_,..b^8^: Empirical coefficients (dimensionless)
t: Duration of irrigation (T)
ρ_b_: Bulk density of soil (ML^−3^)
θ_i_: Initial soil moisture content (L^3^L^−3^)
S: Percentage of sand (%)
S_i_: Percentage of silt (%)
C: Percentage of clay (%)
$\rho_{b1}\;\&\;\rho_{b2}$: Bulk density of soil of upper and lower layer (ML^−3^)
$\theta_{i1}\;\&\;\theta_{i2}$: The initial soil moisture content of the upper and lower layer (L^3^L^−3^)
K_s1_& K_s2_: Saturated hydraulic conductivity of upper and lower layer (L^1^T^−1^)
S_1_& S_2_: Percentage of sand of upper and lower layer (%)
S_i1_& S_i2_: Percentage of silt of upper and lower layer (%)
C_1_& C_2_: Percentage of clay of upper and lower layer (%)
Δθ: Average change in volumetric water content within the wetted zone (L^3^L^−3^)
θ_s_: Moisture content at 0 bar
θ_r_: Residual water content
α: Soil/Shape parameter (dimensionless)
n: Soil/Shape parameter (dimensionless)
CR: Cycle ratio (dimensionless)
V_s_: Wetted soil volume (L^3^)
Z_+_: Vertical upward wetting front (L)
Z: Vertical downward wetting front (L)
z: Emitter installation depth (L)
X_if_: Wetting front for intersection zone in horizontal (L)
Z_if_: wetting front for intersection zone in a vertical direction (L)
t_1_: Intersection time of wetting front in a double source of an emitter (T)
t_2_: Final time of wetting front in the double source of the emitter (T)
R_0_: cylindrical saturated zone with radius (L)
γ: Euler's constant (0.577216)
R_e_: Equivalent radius of the cavity surrounding a single emitter as a function of any soil water pressure head at the source (P)
P: Soil water pressure head at the source
Φ_0_: Soil matric potential (LMT^−2^)
L: Length of ceramic pipe (L)
K_se_: Saturated hydraulic conductivity of the ceramic pipe (LT^−1^)
h: Pressure head (L)
θ: Volumetric soil water content, (L^3^L^−3^)
